# Influence of Software Tool and Methodological Aspects of Total Metabolic Tumor Volume Calculation on Baseline [18F]FDG PET to Predict Survival in Hodgkin Lymphoma

**DOI:** 10.1371/journal.pone.0140830

**Published:** 2015-10-16

**Authors:** Salim Kanoun, Ilan Tal, Alina Berriolo-Riedinger, Cédric Rossi, Jean-Marc Riedinger, Jean-Marc Vrigneaud, Louis Legrand, Olivier Humbert, Olivier Casasnovas, François Brunotte, Alexandre Cochet

**Affiliations:** 1 Department of Nuclear Medicine, Centre Georges-François Leclerc, Dijon, France; 2 Le2i UMR CNRS 6306, Dijon, France; 3 MRI Unit, Centre Hospitalier Régional Universitaire, Hôpital Le Bocage, Dijon, France; 4 Beth Israel Deaconess Medical Center, Boston, Massachusetts, United States of America; 5 Department of Clinical Hematology, Centre Hospitalier Régional Universitaire, Hôpital Le Bocage, Dijon, France; 6 Inserm U866, Labex team, Faculté de Médecine, Université de Bourgogne, Dijon, France; The University of Chicago, UNITED STATES

## Abstract

**Aim:**

To investigate the respective influence of software tool and total metabolic tumor volume (TMTV0) calculation method on prognostic stratification of baseline 2-deoxy-2-[18F]fluoro-D-glucose positron emission tomography ([18F]FDG-PET) in newly diagnosed Hodgkin lymphoma (HL).

**Methods:**

59 patients with newly diagnosed HL were retrospectively included. [18F]FDG-PET was performed before any treatment. Four sets of TMTV0 were calculated with Beth Israel (BI) software: based on an absolute threshold selecting voxel with standardized uptake value (SUV) >2.5 (TMTV0_2.5_), applying a per-lesion threshold of 41% of the SUV_max_ (TMTV0_41_) and using a per-patient adapted threshold based on SUV_max_ of the liver (>125% and >140% of SUV_max_ of the liver background; TMTV0_125_ and TMTV0_140_). TMTV0_41_ was also determined with commercial software for comparison of software tools. ROC curves were used to determine the optimal threshold for each TMTV0 to predict treatment failure.

**Results:**

Median follow-up was 39 months. There was an excellent correlation between TMTV0_41_ determined with BI and with the commercial software (r = 0.96, p<0.0001). The median TMTV0 value for TMTV0_41_, TMTV0_2.5_, TMTV0_125_ and TMTV0_140_ were respectively 160 (used as reference), 210 ([28;154] p = 0.005), 183 ([-4;114] p = 0.06) and 143ml ([-58;64] p = 0.9). The respective optimal TMTV0 threshold and area under curve (AUC) for prediction of progression free survival (PFS) were respectively: 313ml and 0.70, 432ml and 0.68, 450ml and 0.68, 330ml and 0.68. There was no significant difference between ROC curves. High TMTV0 value was predictive of poor PFS in all methodologies: 4-years PFS was 83% vs 42% (p = 0.006) for TMTV0_2.5,_ 83% vs 41% (p = 0.003) for TMTV0_41,_ 85% vs 40% (p<0.001) for TMTV0_125_ and 83% vs 42% (p = 0.004) for TMTV0_140_.

**Conclusion:**

In newly diagnosed HL, baseline metabolic tumor volume values were significantly influenced by the choice of the method used for determination of volume. However, no significant differences were found in term of prognosis.

## Introduction

Metabolic tumor volume (MTV) calculated on 2-deoxy-2-[18F]fluoro-D-glucose ([18F]FDG) positron emission tomography (PET) is a promising parameter for prognostic stratification in various tumors and could be used to drive patient management in a risk adapted strategy [1]. Despite promising results, methodological aspect of MTV determination is still unclear. Many MTV calculation methods are available and are being used in research but as yet no consensus has been reached. Available methods use different levels of complexity with different points of strength and weakness. In recently published papers, most of the MTV determination used an approach based on standardized uptake value (SUV) thresholds. The two usual thresholds are based on a fixed threshold (usually SUV>2.5 [2]) or a relative threshold (usually SUV> 41% of the SUVmax of the tumor [3]). Other methodologies have been proposed: adaptive threshold that allows consideration of the background activity or more sophisticated methods such as fuzzy C mean (FCM) and fuzzy locally adaptive Bayesian (FLAB) algorithms [4]. These methods providing different MTV measurements, comparative studies are needed to evaluate the inter-method variations and to establish the method of choice [5]. In our institution we recently performed a retrospective single center study that showed a strong and independent prognostic value of total metabolic tumor volume (TMTV0) calculated on baseline [18F]FDG PET/CT for newly diagnosed Hodgkin lymphoma [6]. Using a relative threshold (>41% SUVmax) we determined that 225 ml was the best TMTV0 cut-off to predict patient outcomes. This strong prognosis value was confirmed by another retrospective study that used a fixed threshold (SUV>2.5) and found a different cut-off (198 ml) [7]. The comparison of these two studies is limited by differences in the MTV methodology used and in patient characteristics (all Ann Arbor stages versus early stage only). The high prognosis value shown in these two studies underlines the clinical importance of TMTV0 and could be useful for patient management in further clinical studies. Before implementing TMTV0 in clinical trials, methodological data are needed to determine the best methodology for TMTV0 calculation. Without the availability of an adapted gold standard, only a comparative study with a single dataset of patients could be used to find the best prognosis value of different TMTV0 methodologies.

The choice of the software tool is also a potential source of bias in the determination and reproducibility of TMTV0, given the fact that available commercial software includes different shapes of volume of interest (VOI) drawing (predefined or irregular) and different levels of display and visual control of TMTV0 calculation.

The aim of this study was to compare the TMTV0 assessment, prognosis strength and reproducibility of several TMTV0 calculation methods determined on baseline [18F]FDG PET/CT in patients with newly diagnosed Hodgkin lymphoma. For that purpose we also developed a new free and widely available software tool for TMTV0 calculation and investigated the influence of software design in the TMTV0 assessment.

## Material and Methods

### Patients

Using a previously published dataset of patients [6], we retrospectively analyzed 59 consecutive patients with a first diagnosis of classical HL, excluding nodular lymphocyte-predominant lymphoma, referred to Dijon University Hospital between January 2007 and January 2010. All the patients provided a written informed consent. The study procedures were in accordance with the ethical standards of the committees with responsibility for human experimentation and with the Helsinki Declaration of 1975, as revised in 2008. In case of a minor patient, the written consent of the legal guardian and the oral consent of the patient was required (the written consent of the minor patient was not mandatory according to the French legislation). The oral consent was reported in the medical record. The whole procedure for this study was approved by the ethical committee (comité de protection des personnes Est I, France). All patients had negative serology for HIV. The diagnosis of classical HL was mainly based on lymph node histology and classified according to the 2008 WHO classification of hematologic malignancies [8]. HL was considered unclassified when only a biopsy of extra nodal tissue was available.

Staging of the disease was performed in accordance with the Ann Arbor classification using enhanced computed tomography (CT) scan of the neck, thorax abdomen and pelvis, and bone marrow biopsy. A single tumor mass greater than 10 cm on enhanced CT was considered a bulky tumor.

Patients’ characteristics are listed in Table 1.

**Table 1:** Patients’ characteristics.

Characteristics		Patients: N (%)
Median age at diagnosis (years)		35.5 (16–76)
Histological type		
	Lymphocyte rich	5 (9)
	Mixed cellularity	7 (12)
	Nodular sclerosis	45 (76)
	Unclassified	2 (3)
Ann Arbor Stage		
	I	5 (8)
	II	17 (29)
	III	10 (17)
	IV	27 (46)
Bulky Tumor (mass>10cm)		9 (15)
IPS score		
	< 2	23 (39)
	≥ 3	36 (61)

### Treatment and patients’ outcomes

Patients were treated according to the LYmphoma Study Association (LYSA) (formerly Groupe d’Etude des Lymphomes de l’Adulte (GELA)) recommendations: patients with stage I and II disease received four to six cycles of an anthracycline-based chemotherapy regimen, followed by 20 to 36 Gy of involved-field radiotherapy; patients with stage III and IV disease received eight cycles of anthracycline-based chemotherapy.

Tumor response was assessed using the revised Cheson criteria [9] at the end of treatment, except for patients with progressive disease who were evaluated at the time of progression. Fifty-four patients (92%) achieved at least a partial response (88% complete response) and five patients (8%) had progressive disease. Five patients (8%) relapsed, with a median time to relapse of 24 months (range, 6 to 36 months). Five patients (8%) died: three from HL progression, one from hepatocellular cancer, and one from bleomycin-related pulmonary fibrosis. The median follow-up was 50 months (range, 22 to 71 months).

### PET acquisition

PET was performed at baseline (PET0) in accordance with the policy of the Hematology Department of Dijon Hospital since 2005, which requires systematic PET evaluation for [18F]FDG avid lymphoma.

Whole-body PET was acquired sequentially using a dedicated PET/CT system (Gemini GXL or Gemini TOF, Philips Medical Systems, Eindhoven, The Netherlands). CT scans were used for anatomic registration and also for attenuation correction. Emission data were corrected for dead time, random and scatter coincidences and attenuation before reconstruction with the RAMLA iterative method. The image voxel counts were calibrated to activity-concentration (Bq/mL) and decay-corrected using the time of tracer injection as the reference.

All of the patients were instructed to fast for at least 6 hours before the injection of [18F]FDG. Serum glucose levels were measured using the hexokinase method. Whole-body emission and transmission scans were acquired in the 3D mode, 60 minutes after the i.v. administration of 3 (Gemini TOF) or 5 (Gemini GXL) MBq/kg of [18F]FDG. Non-contrast-enhanced CT images were acquired before PET data acquisition. The CT, PET, and co-registered PET/CT images were reviewed in the axial, coronal and sagittal planes along with maximum intensity projection (MIP) whole-body images.

### Software tool

We developed a new software tool based on Beth Israel plugin for FIJI [10]. This shareware from the Beth Israel Deaconess Medical Center, Division of Nuclear Medicine and Molecular Imaging is available at http://sourceforge.net/projects/bifijiplugins/ as a free plug-in for FIJI [11] (Image J distribution).

This software handles PET/CT fusion and display (multi-planar reconstruction and maximum intensity projection). We added MTV calculation capability based on absolute SUV threshold (ex >2.5 SUV) or relative threshold (ex >41% SUVmax).

This software is available under General Public License and can be used with all operating systems (Windows, OSX or GNU/Linux).

For software validation, blinded calculation of TMTV0 was made with Beth Israel plugin and compared to the TMTV0 results to a commercial software, using Keosys software (FDA 510k clearance) as reference.

Two validations were made. First, quantifications were made on the NEMA IEC body phantom (Data Spectrum Corporation, Hillsborough, NC) to check the absolute reproducibility of the two packages. Then, in a real clinical TMTV0 determination using the previously described patients population with comparison of previously published [6] TMTV0 values calculated with the commercial software. The prognosis strength of the TMTV0 values as calculated by the two software packages was also compared.

### TMTV0 assessment

To assess the TMTV0, all of the images were independently reviewed by two experienced nuclear medicine physicians blinded to the patients’ outcomes.

To determine TMTV0, volume of interest (VOI) were drawn around each focus of [18F]FDG uptake on pre-treatment PET/CT. In each VOI, four TMTV0 determinations were performed: absolute threshold selecting voxel with SUV>2.5 (TMTV0_2.5_), per-lesion relative threshold of 41% of the SUV_max_ (TMTV0_41_) and per-patient adapted thresholds selecting voxel over 125% and 140% of the SUVmax of liver background (TMTV0_125_ and TMTV0_140_). Liver background was measured by drawing a circle VOI of 20 mm of diameter in the base of the hepatic dome.

**Fig 1:**
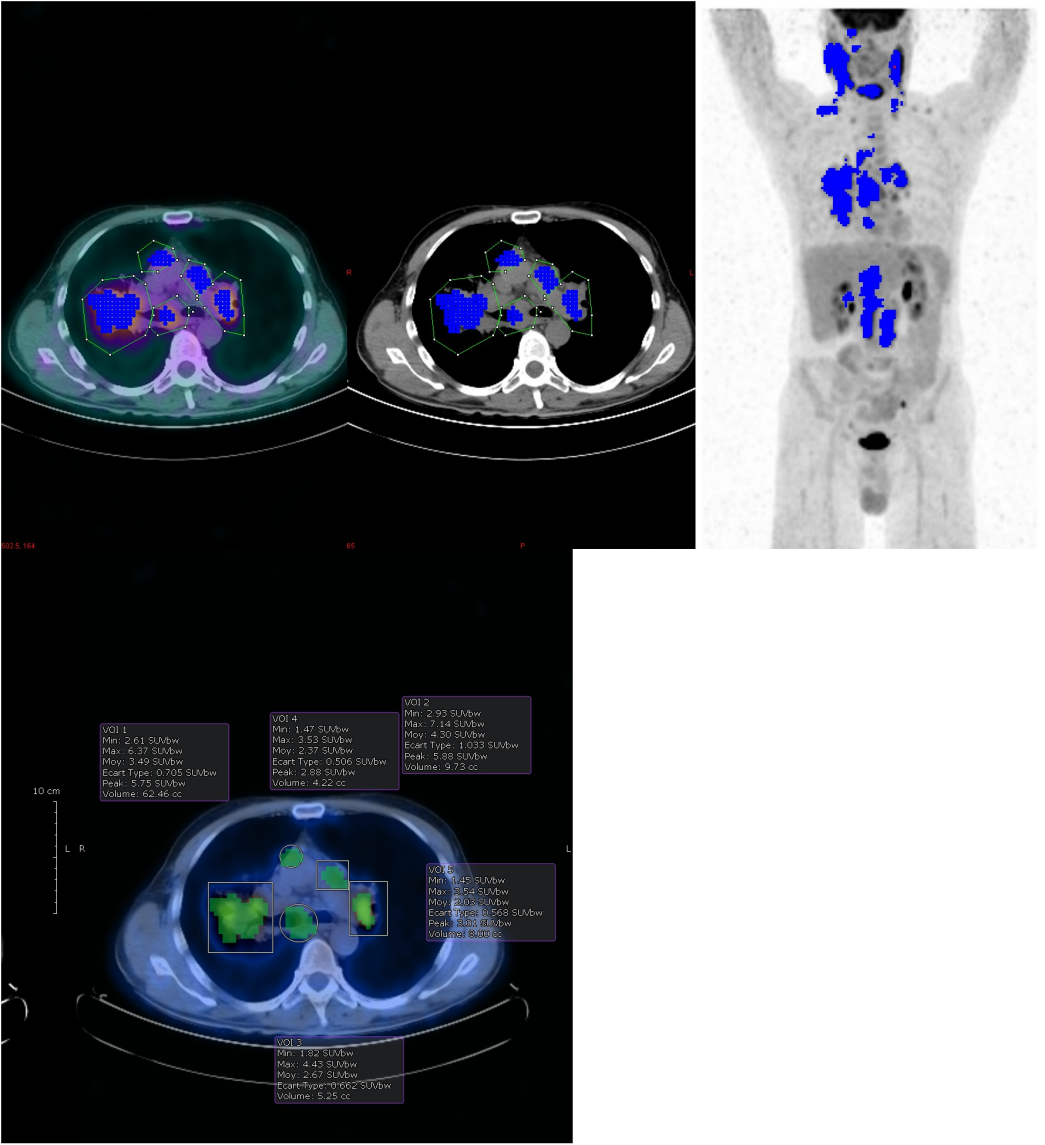
TMTV0 calculation example and VOI drawing depending on software. TMTV0_41_ calculation example. VOIs were drawn with Beth Israel plugin (a) and a commercial software (b). The two packages allow different VOIs definition using irregular or predefined shapes (see discussion).

All calculations used the same VOI definitions (Fig 1). During VOI drawing, in case of heterogeneous uptake, high local uptake was isolated in a separate VOIs to avoid underestimation of the tumor volume in the relative SUVmax threshold approach. TMTV0 calculations were visually verified in all methodologies, the investigators checked that voxels included in each TMTV0 calculation had no physiological background (the voxels which were included were highlighted by the software).

Extra-nodal involvement was considered in the volume calculation according to the following rules: the liver, lung and bone marrow were considered involved only in cases of focal uptake and the volume of each individual hyper metabolic lesion was computed in a separate VOI; homogeneous bone marrow uptake was not included in the tumor volume; spleen involvement was considered in cases of focal uptake or diffuse uptake higher than 150% of the liver background.

All the individual lesion volumes were added together to calculate TMTV0.

### Statistical analysis

All quantitative data were expressed as mean ± standard deviation (SD) or median (first quartile–third quartile) as appropriate, and qualitative data were expressed as numbers and percentages.

The correlation between TMTV0 values was computed using Pearson coefficient and the differences were assessed using Bland-Altman analysis and Student's t-test.

TMTV0 assessment was also compared for their respective prognostic value. Due to the high correlation value of TMTV0 calculations between readers (see further), the prognosis value was set using the results from only one reader. The prognostic relevance of each TMTV0 calculation was compared using a pairwise comparison of ROC curves[12]. Best TMTV0 cut-off was determined by applying the receiver operating characteristics approach, based on their ability to predict treatment failure (progression or relapse of HL) with the best sensitivity and specificity according to the Youden index [13]. p values <0.05 were considered statistically significant.

For the software validation we compared the values of TMTV0_41_ obtained with Beth Israel plugin for FIJI with those obtained with the commercial software on the NEMA IEC body phantom and the dataset of 59 patients. Statistical analysis compared the quantitative data and the prognosis strength of the two determinations of TMTV0.

To compare TMTV0 assessment of each methodology, TMTV0 were also compared with a quantitative and prognosis analysis. Reproducibility analysis was made using a quantitative comparison of TMTV0 calculations (Pearson coefficient and Bland-Altman analysis) and kappa coefficient to calculate inter observer agreement using the optimal TMTV0 cut-off.

For each methodology, survival functions of subgroups of patients defined by the TMTV0 were estimated using the Kaplan-Meier product limit method and compared using the log-rank test;

Progression-free survival (PFS) was analyzed according to the TMTV0 values. The PFS was defined as the time from the beginning of treatment until disease progression, relapse or death (from any cause) or the date of last follow-up. Finally, multivariate Cox regression analysis was performed to test for predictors of PFS. All parameters in Table 1, and TMTV0 values were tested by univariate analysis. For multivariate analysis, each TMTV0 parameter was tested against the international prognostic score[14] (IPS) >2 and Bulky disease, resulting in 4 models.

## Results

### Software comparisons

On the phantom images, Beth Israel plugin and the commercial software showed exactly same TMTV0_41_ values in the six hot spheres (0% difference). The two packages also provide exactly the same values of SUVmax and SUVmean.

On the patient population, correlation of the TMTV0_41_ value was excellent (r = 0.96 p<0.0001). The median TMTV0_41_ was respectively 161 and 117ml. The mean TMTV0_41_ was 243ml for Beth Israel plugin and 207ml for the commercial software with significantly higher value with Beth Israel plugin compared to the commercial software +36ml (CI [16.4–59.4], p<0.001).

**Fig 2:**
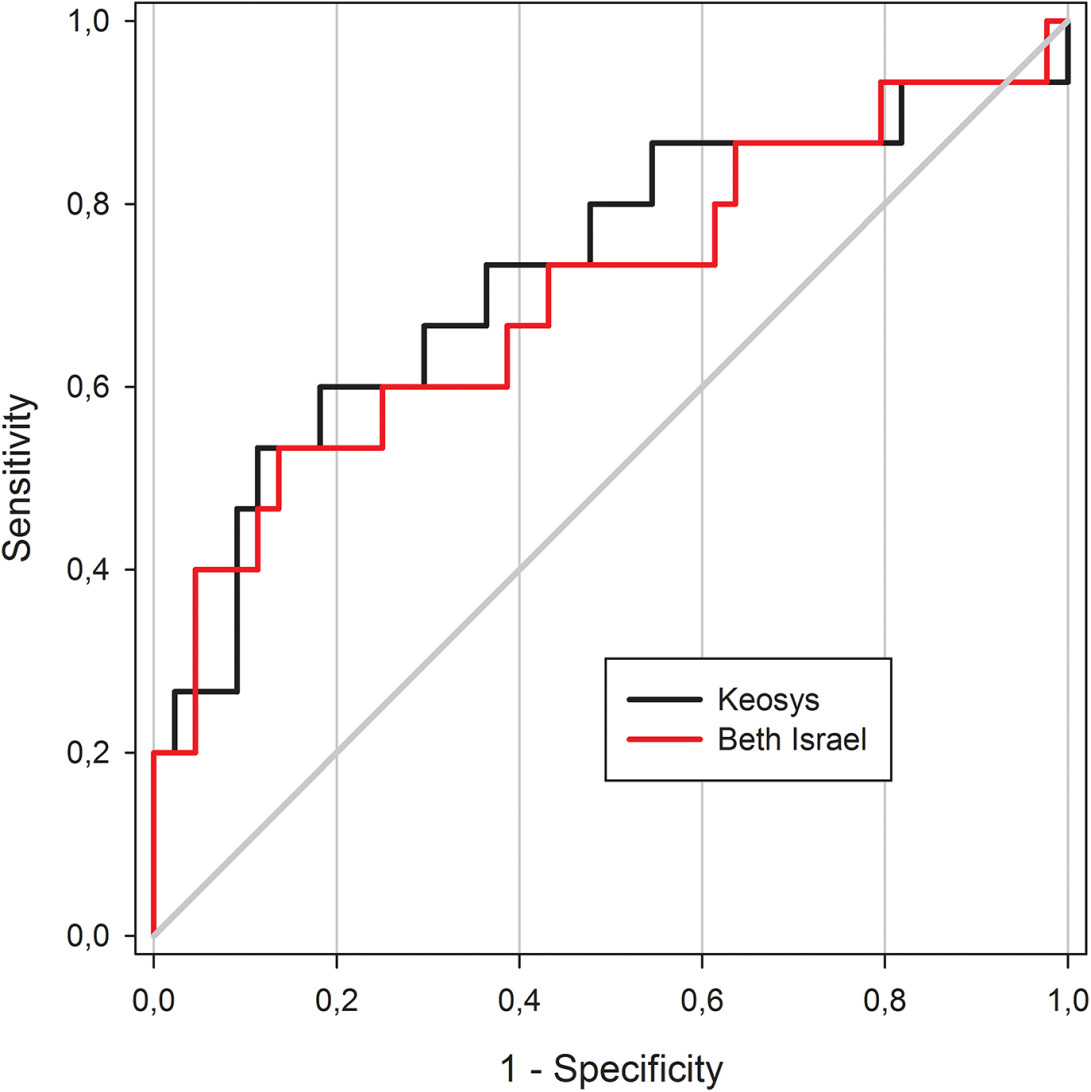
ROC curves according to software ROC curves using TMTV0_41_ with Beth Israel plugin (red line) and the commercial software (black line).

Concerning the prognosis value, no significant differences between the AUC could be seen using the two TMTV0_41_ assessments (Fig 2). AUC was 0.70 for Beth Israel plugin and 0.72 for the commercial software (p = 0.19). The optimal cut-off to predict patient outcomes was 313ml for Beth Israel plugin and 225ml for the commercial software. Using those cut-offs both packages were able to predict the PFS with a 4 years PFS of 83% vs 41% (p = 0.003) for Beth Israel plugin and 85% vs 42% (p = 0.001) for the commercial software.

### Comparison of TMTV0 methodologies

**Fig 3:**
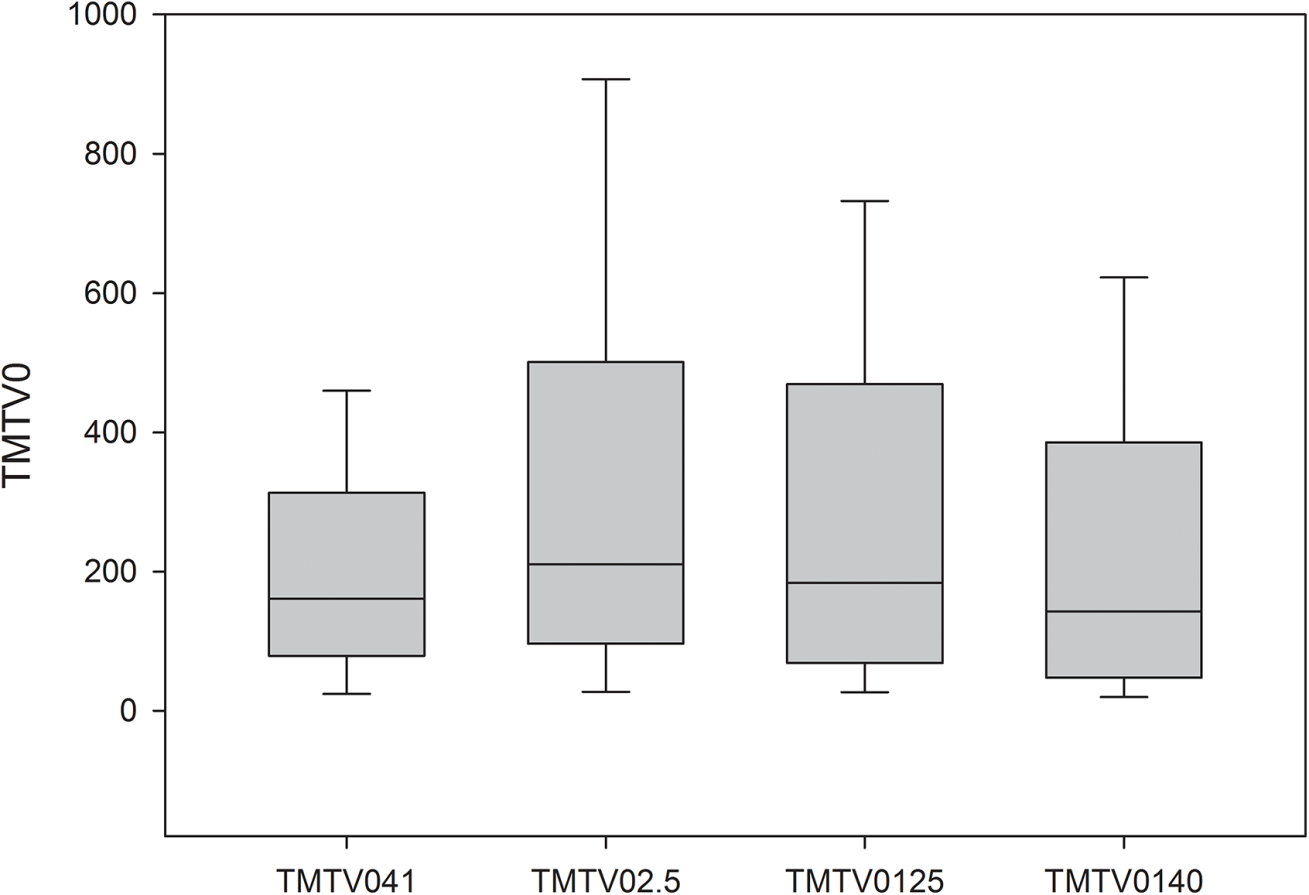
TMTV0 distribution according to each methodology. TMTV0 distribution with median (black lines), 25 to 75 percentile (grey boxes), 10 and 90 percentile (edges) according to each TMTV0 methodology.

**Fig 4:**
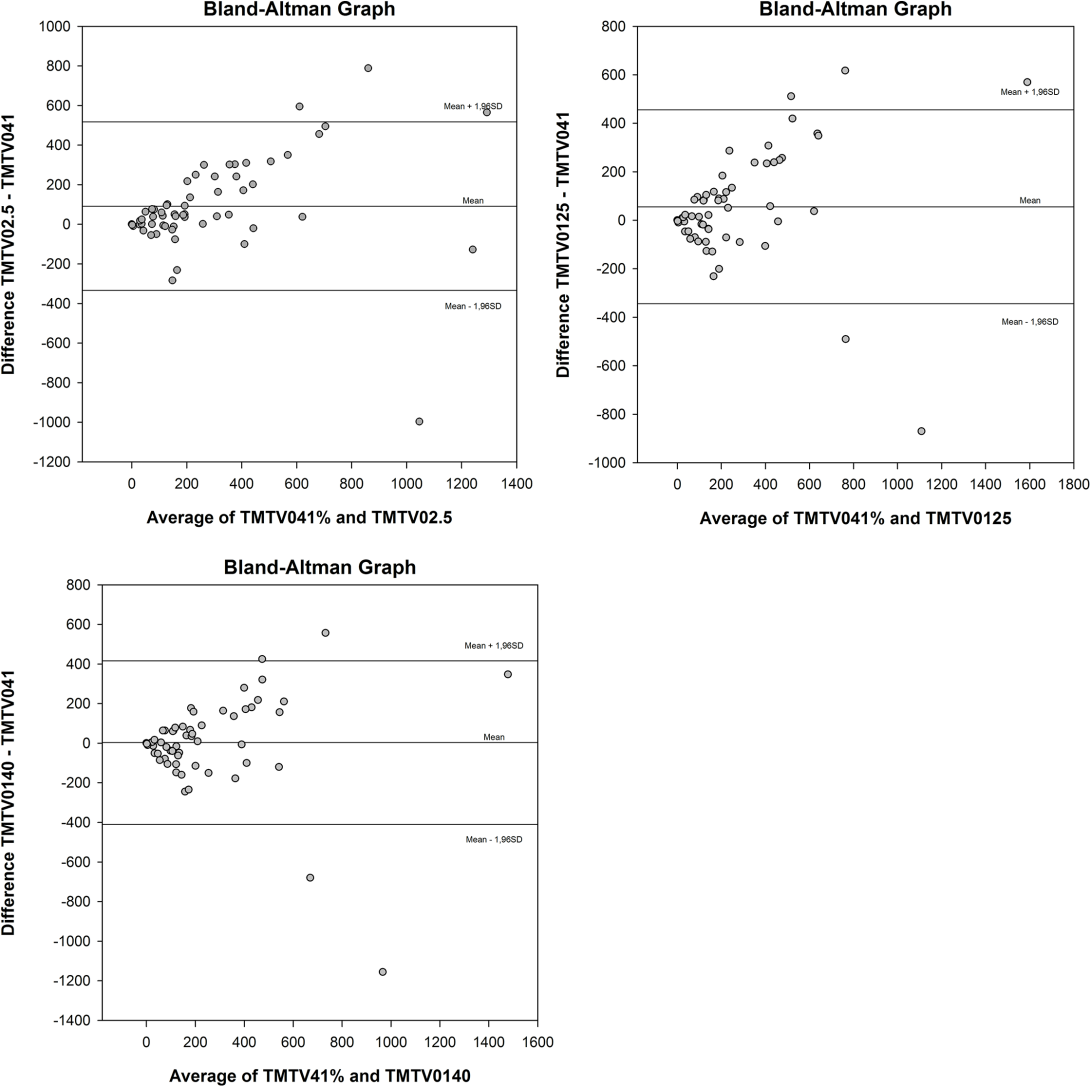
Bland Altman analysis of different TMTV0 methodologies. Bland-Atman analysis comparing TMTV0 values of TMTV0_2.5_ (a), TMTV0_125_ (b) and TMTV0_140_ (c) to TMTV0_41_. Mean bias and limits of agreements are represented by solid lines.

The median value of TMTV0_2.5,_ TMTV0_41,_ TMTV0_125_ and TMTV0_140_ were respectively 210 (range 0-1574ml), 160 (range 0-1544ml), 183 (range 0-1874ml) and 143ml (range 0-1651ml). The mean values were respectively 335, 243, 299 and 247ml. The distribution of respective TMTV0 values and Bland Altman analysis are represented in Figs 3 and 4.

The TMTV0_2.5_ showed significant higher value than TMTV0_41_: +92ml CI [28; 154] p = 0.005. No significant difference was found between TMTV0_41_ and TMTV0_125_ or TMTV0_140_ (respectively +56ml, CI [-4; 114], p = 0.06 and +4ml, CI [-58; 64], p = 0.9).

**Fig 5:**
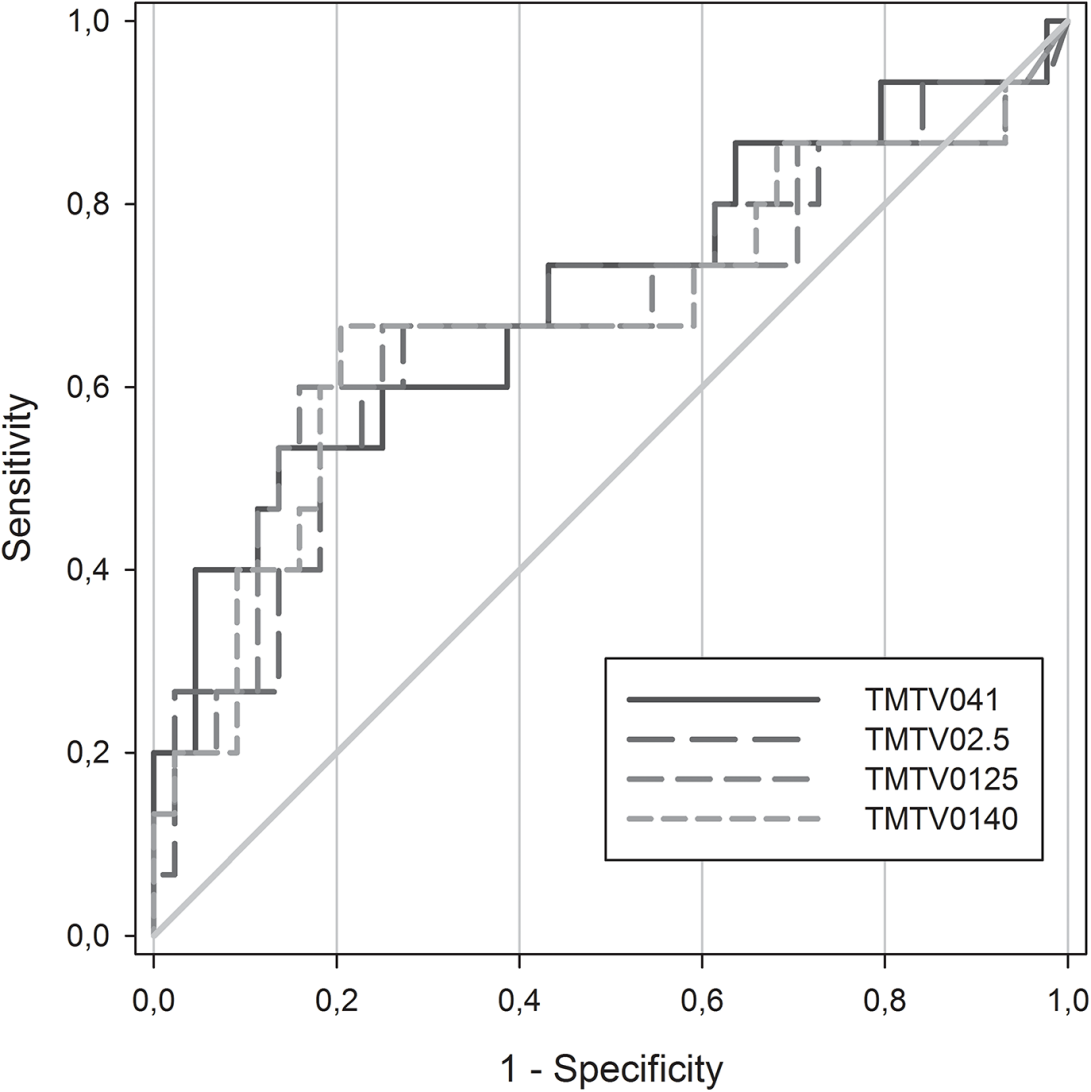
ROC curves according to methodologies. ROC curves comparison for each methodology. The respective optimal cut-off and area under curve of TMTV0_41_, TMTV0_2.5_, TMTV0_125_ and TMTV0_140_ were respectively 313ml and 0.70, 432ml and 0.68, 450ml and 0.68, 330ml and 0.68.

The AUC were not significantly different in any of the methodologies (Fig 5). The respective AUC of TMTV0_2.5,_ TMTV0_41,_ TMTV0_125_ and TMTV0_140_ were respectively 0.68, 0.70, 0.68 and 0.68 (p>0.6 for each pairwise comparison). The best TMTV0 cut-off was 432 ml for TMTV0_2.5,_ 313ml for TMTV0_41,_ 450ml for TMTV0_125_ and 330ml for TMTV0_140._ The sensitivity, specificity, positive predictive value and negative predictive value were respectively 53%, 82%, 50%, 84% for TMTV0_2.5_, 53%, 91%, 57%, 92% for TMTV0_41_, 60%, 81%, 53%, 86% for TMTV0_125_ and 53%, 82%, 50%, 84% for TMTV0_140._

**Fig 6:**
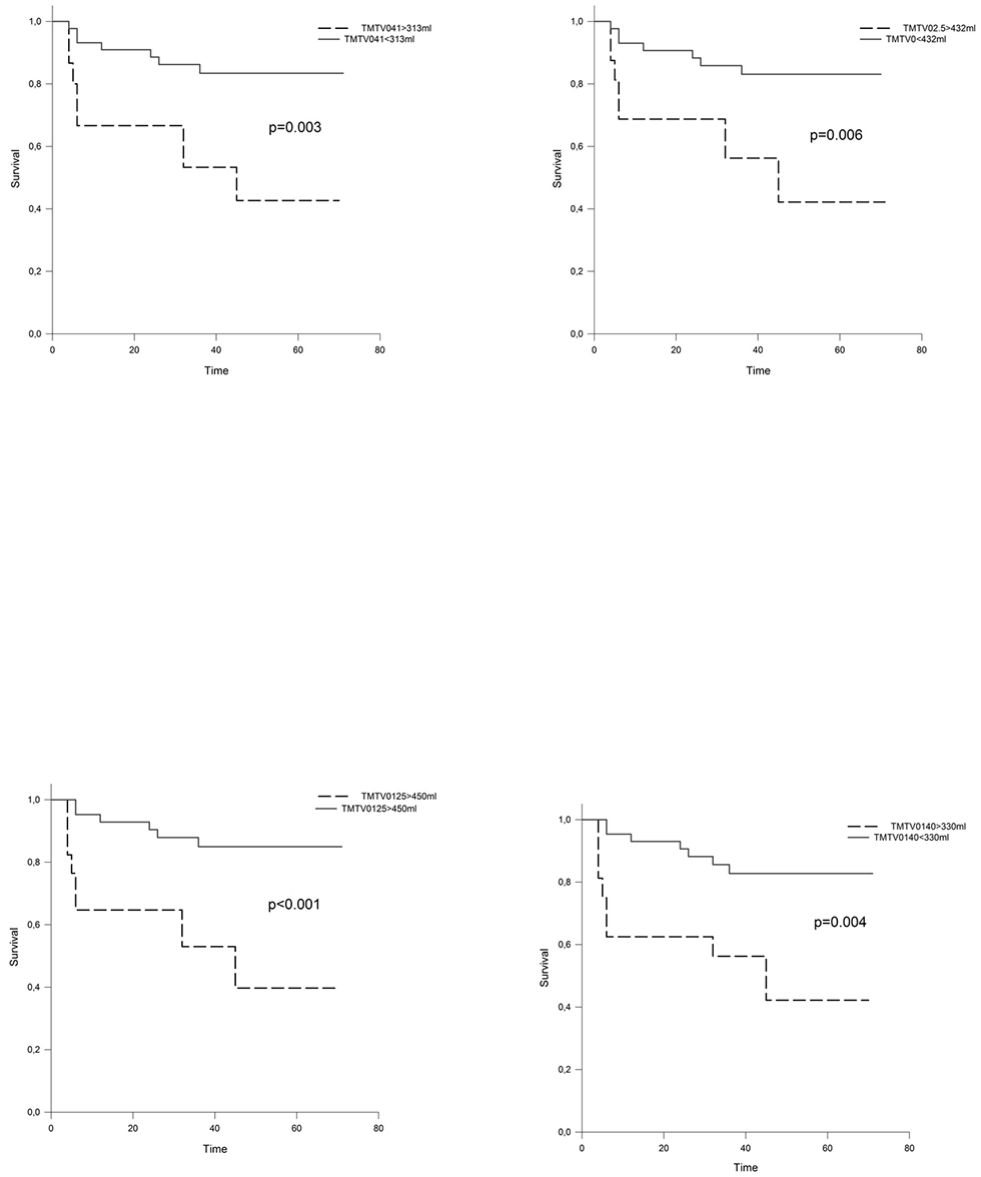
PFS survival according to methodologies. PFS survival analysis according to TMTV0_2.5_ (a), TMTV0_41_ (b), TMTV0_125_ (c), and TMTV0_140_ (d).

All the tested TMTV0 methodologies were predictive of PFS (Fig 6). Patients having a high TMTV0 according to the previously defined cut-off for each methodology had a significant poorer prognosis. 4-years PFS was 83% vs 42% (p = 0.006) for TMTV0_2.5,_ 83% vs 41% (p = 0.003) for TMTV0_41,_ 85% vs 40% (p<0.001) for TMTV0_125_ and 83% vs 42% (p = 0.004) TMTV0_140_. Results of the Cox proportional hazards regression models for prediction of PFS are reported in Table 2. Only factors having a p value less than 0.1 in univariate analysis on the log-rank test were included. The TMTV0 remained an independent predictor of event, whatever the method of computation used, even when adjusted for IPS and presence of bulky disease.

**Table 2:** Multivariate analysis of each TMTV0 methodology to predict PFS.

Multivariate analysis		HR (95% CI)	*p*
Model 1			
	TMTV0_2.5_ > 432ml	3.24 (1.15–9.13)	0.026
	IPS>2	2.38 (0.66–8.62)	0.186
	Bulky	1.51 (0.48–5.81)	0.478
Model 2			
	TMTV0_41_ > 313ml	3.36 (1.77–9.66)	0.025
	IPS>2	2.14 (0.58–7.94)	0.251
	Bulky	1.74 (0.60–5.09)	0.309
Model 3			
	TMTV0_125_ > 450ml	4.17 (1.45–12.0)	0.008
	IPS>2	2.27 (0.63–8.26)	0.212
	Bulky	1.53 (0.52–4.57)	0.439
Model 4			
	TMTV0_140_ > 330ml	3.50 (1.25–9.84)	0.017
	IPS>2	2.47 (0.70–8.90)	0.164
	Bulky	1.75 (0.61–5.09)	0.298

### Inter observer reproducibility

The Pearson coefficient for the two determinations of TMTV0 was 0.99 for TMTV0_2.5_, 0.91 for TMTV0_41,_ 0.98 for TMTV0_125_ and 0.98 for TMTV0_140_ (p<0.001). Kappa coefficient was respectively 0.96, 0.77, 0.96 and 0.91.

In Bland-Altman analysis the bias was -6.83 ml (SD = 29.69, CI [-14,56; 0,90]) for TMTV0_2.5_, 15.50 ml (SD = 123.93 CI [-16,773; 47,7689]) for TMTV0_41_, -51.48 ml (SD = 89.96 CI [-74,9143; -28,0634]) for TMTV0_125_, -40.11 ml (SD = 67.97 CI [-57,81; -22,41]) for TMTV0_140_.

## Discussion

In Hodgkin lymphoma, baseline total metabolic tumor volume (TMTV0) as determined by [18F]FDG PET has been recently demonstrated to have a strong prognosis value [6,7,15], and could be implemented in clinical trials to introduce risk adapted strategy for patient management. However, the choice of TMTV0 calculation methodology is still being debated and needs to be standardized before reaching a clinical protocol. The two available studies about TMTV0 in Hodgkin lymphoma were based on different TMTV0 methodology (SUV>2.5 and 41% threshold). Thus, we designed this new study to compare different methodologies for determination of TMTV0 in the same dataset of patients and to calculate their impact on the prognosis value and cut-off determination.

The absolute threshold using SUV>2.5 is the simplest determination and is widely available in commercial software. This absolute cut-off allows a simple volume calculation using a fixed threshold to select voxel in all VOIs and may reduce the inter-observer variability in calculating the TMTV0 value. Despite its advantages, this absolute SUV based approach is limited by its lack of reproducibility of SUV values, likely influenced by biological and technological factors [16] leading to a higher variability depending on PET acquisition protocol and devices.

The relative threshold using 41% of the SUVmax value has been validated in a phantom study and in Hodgkin lymphoma [17,18] and is also recommended by the EANM[17]. Using a threshold of per lesion SUVmax value avoids the reproducibility issues of an absolute SUV based approach but introduce a VOI drawing variability. The cut-off calculation being based on per VOI SUVmax value, each uptake needs to be carefully included in a single VOI which can be difficult to set in case of large and heterogeneous uptakes typical in Hodgkin lymphoma [19]. The two main pitfalls using relative threshold are encountered in the cases of heterogeneous or low uptake. In the case of low uptake the 41% of SUVmax value could be lower than the background activity, which would overestimate the calculated volume by selecting voxel in the background. In the case of heterogeneous uptake, the metabolic tumor volume could be underestimated in case of a locally high uptake value, excluding the less metabolically active part of the tumor.

In this study we also introduced a per-patient adapted threshold based on liver background. The liver background is commonly used as reference to define a significant uptake and is used in the 5-point scale [20] to define a residual tumor uptake in response assessment in Hodgkin lymphoma. We used 125% and 140% of liver background as threshold to define malignant uptake, those thresholds were chosen to select significant uptake according to visual analysis [21]. The first advantage of this adapted per-patient absolute threshold is to avoid the heterogeneity issue, the same SUV value threshold being used in all VOIs. The second advantage is the adaptation of the threshold value to each patient, avoiding the reproducibility issue of a fixed SUV value threshold [16,22] that could be an important limitation in multicenter trial. The main limitation of this methodology could be the heterogeneity of [18F]FDG uptake in the liver that could lead to measurement variability but probably with low impact, the liver heterogeneity being rather small [23].

To validate the best methodology, the reproducibility of TMTV0 is an important parameter to consider. In this study, fixed or patient adapted to liver SUV cut-off showed better reproducibility than TMTV0_41_. Best reproducibility was found using a fixed SUV cut-off of 2.5 probably because of the small variation in liver background measurement. However, the reproducibility of fixed and liver based cut-offs remains very good with excellent correlation and kappa coefficient. Using 41% of SUV threshold, kappa coefficient was lower (0.77), with 5 patients misclassified between the two physicians. In two of these five patients discordance was due to different interpretation of bone marrow uptake. These two patients had heterogeneous bone marrow uptake with low contrast, which was totally included by one physician and partially included by another. After consensus for these two patients the kappa coefficient of TMTV0_41_ was 0.85. Bone marrow uptake in Hodgkin lymphoma presents a large range of patterns from diffuse uptake to heterogeneous uptake with a large range of contrast. The clinical significance of these bone marrow patterns is still unknown. In this study we didn’t include diffuse uptake that could be more likely related to an inflammatory uptake. Heterogeneous uptakes were considered as bone marrow involvement but could lead to different interpretations in case of small heterogeneities. The use of 41% of SUVmax increases those differences of interpretation due to the high number of voxels included in those cases of low contrast. The prognostic significance of bone marrow patterns need to be clarified to define more precisely the TMTV0 calculation.

In our results we found significantly higher TMTV0 values using TMTV0_2.5_ compared to TMTV0_41_. TMTV0_2.5_ also provided the higher TMTV0 optimal cut-off in our population (432ml). Two main reasons could explain the higher TMTV0_2.5_ values: First, using this low SUV cut-off, voxel outside of the tumor could be selected by partial volume effect on the border of high uptake tumors like Hodgkin lymphoma [19]. Then, voxel in the physiological background of some tissues or inflammatory uptake could also reach this threshold [24] and be counted in the TMTV0_2.5_.

Using TMTV0_2.5_ in Hodgkin Lymphoma, Song et al. [7] found a best cut-off at 198ml which was lower than our previous study finding an optimal cut-off at 225ml using TMTV0_41_ [6]. The optimal cut-off calculated using TMTV0_2.5_ in our population was 432ml which is clearly higher than the cut-off reported by Song et al. [7]. These differences could be due to patient selection in the determination of the optimal cut-off. Song et al. selected only early stage (I and II) whereas all stages were included in our previous paper. In our previous paper we also reported significant higher TMTV0_41_ values for stage Ann Arbor IV than other stages. The differences of the two studies may be related to two adverse effects: The choice of a 2.5 SUV cut-off leads to higher TMTV0 values but the determination of TMTV0_2.5_ in a population with only early Ann Arbor stages results in the inclusion of patients with a lower tumor burden in comparison to our series. The difference in patient selection may be particularly important due to the extensive fraction of our patients having an Ann Arbor stage IV (46%) and could lead to major differences of patient outcomes between the two populations. Considering the TMTV0 prognosis value, despite significant changes in TMTV0 values we were not able to find significant differences in the prognosis strength for these two determinations. Those methodological aspects need to be explored in further studies. In this study, per-patient adapted liver methodologies have shown a good reproducibility, a high prognosis value to predict PFS and were in univariate as in multivariate analysis, slightly better than the other methodologies, even if it was not statistically significant. Liver based approaches could be proposed as a valuable methodology due to their valuable prognosis strength, reproducibility and the smaller sensitivity to heterogeneous uptakes that may solve the main limitations of both TMTV0_2.5_ and TMTV0_41_ for TMTV0 calculation.

To minimize the time consumption of TMTV0 calculation, we built and validated a new software tool. When compared with the commercial software, TMTV0_41_ values of the two packages showed similar prognosis values and a very good correlation. However, a significant difference on TMTV0_41_ values was found leading to slightly superior values with Beth Israel plugin compared to the commercial software. These differences lead to a different cut-off value with our new software (313 ml vs 225ml), that could be due to the higher TMTV0 values with Beth Israel software and the difficulty to define a clear best TMTV0 cut-off in this population with small variations of Youden index between 289 and 334 ml (Youden index from 0.35 to 0.39). Despite changes in optimal cut-off, the 225ml cut-off in TMTV0 values using Beth Israel software still achieve significant prognosis value (log rank test p = 0.03). The differences in TMTV0_41_ values in the patient datasets are probably related to changes in VOI definition due to differences in software design. In our new software, we simplified VOI definition, added visuals controls and implemented a management of VOI overlapping. Using Beth Israel plugin, we were able to draw more VOIs to avoid underestimation of TMTV0_41_ in cases of heterogeneous uptake. This study illustrates the importance of software design in the TMTV0 calculation that need to be simplified and optimized to reach a routine use. Beth Israel plugin for FIJI provides an accurate free tool for TMTV0 calculation that could be used for many purposes and could implement new features and optimizations to build a collaborative software tool for PET/CT processing.

This study also presents some limitations: First, other TMTV0 methodologies have been proposed and were not evaluated in this study, in particular the most sophisticated methods like fuzzy locally adaptive Bayesian [25]. Even if those methodologies have shown interesting prognosis value in solid tumors [26], the prognosis value in Hodgkin lymphoma is still unknown. The availability of those methodologies is also an important parameter to consider, those methods still not being available in most of commercial software. Then, this study being single centric, this data also need to be evaluated in various conditions of PET/CT acquisition. A large multicenter study would emphasize the respective strengths and pitfalls of each methodology with a closer assessment of a routine application.

## Conclusions

Before clinical applications TMTV0 calculations will need to be standardized to be used in patient’s management. Even without significant difference on the prognosis strength, this study illustrates the influence of methodological and software choices that need to be taken in consideration for clinical protocols implementation.

## Supporting Information

S1 Table Supporting Information. Individual data.
(CSV)

## Abbreviations

[18F]FDG: 18Fluorodeoxyglucose or 2-Deoxy-2-[18F]fluoroglucose
AUC: Area Under Curve
CT: Computed Tomography
FCM: Fuzzy C mean
FLAB: Fuzzy Locally Adaptive Bayesian
HL: Hodgkin Lymphoma
MTV: Metabolic Tumor Volume
PET: Positron Emission Tomography
PFS: Progression-Free Survival
SUV: Standardized Uptake Value
TMTV: Total Metabolic Tumor Volume
VOI: Volume Of Interest
